# Revisiting the Basic Reproductive Number for Malaria and Its Implications for Malaria Control

**DOI:** 10.1371/journal.pbio.0050042

**Published:** 2007-02-20

**Authors:** David L Smith, F. Ellis McKenzie, Robert W Snow, Simon I Hay

**Affiliations:** 1 Fogarty International Center, National Institutes of Health, Bethesda, Maryland, United States of America; 2 Malaria Public Health and Epidemiology Group, Centre for Geographic Medicine, Kenya Medical Research Institute, Nairobi, Kenya; 3 Centre for Tropical Medicine, John Radcliffe Hospital, University of Oxford, Oxford, United Kingdom; 4 Spatial Epidemiology and Ecology Group, Department of Zoology, University of Oxford, Oxford, United Kingdom; Pennsylvania State University, United States of America

## Abstract

The prospects for the success of malaria control depend, in part, on the basic reproductive number for malaria, *R*_0_. Here, we estimate *R*_0_ in a novel way for 121 African populations, and thereby increase the number of *R*_0_ estimates for malaria by an order of magnitude. The estimates range from around one to more than 3,000. We also consider malaria transmission and control in finite human populations, of size *H*. We show that classic formulas approximate the expected number of mosquitoes that could trace infection back to one mosquito after one parasite generation, *Z*_0_(*H*), but they overestimate the expected number of infected humans per infected human, *R*_0_(*H*). Heterogeneous biting increases *R*_0_ and, as we show, *Z*_0_(*H*), but we also show that it sometimes reduces *R*_0_(*H*); those who are bitten most both infect many vectors and absorb infectious bites. The large range of *R*_0_ estimates strongly supports the long-held notion that malaria control presents variable challenges across its transmission spectrum. In populations where *R*_0_ is highest, malaria control will require multiple, integrated methods that target those who are bitten most. Therefore, strategic planning for malaria control should consider *R*_0_, the spatial scale of transmission, human population density, and heterogeneous biting.

## Author Summary

Each year malaria results in more than a million deaths. Controlling this disease involves understanding its transmission. For all infectious disease, the basic reproductive number, *R*_0_, describes the most important aspects of transmission. This is the expected number of hosts that can trace their infection directly back to a single host after one disease generation. For vector-borne diseases, such as malaria, *R*_0_ is given by a classic formula.

We made 121 estimates of *R*_0_ for Plasmodium falciparum malaria in African populations. The estimates range from around one to over 3,000, providing much higher estimates than previously thought. We also show that in small human populations, *R*_0_ approximates transmission when counting infections from mosquito to mosquito, but overestimates it from human to human.

Previous studies showed that transmission is amplified if some humans are bitten more than others. We confirm that such heterogeneous biting amplifies transmission counting from mosquito to mosquito, but it can also dampen transmission counting from human to human. Humans who are bitten most both infect a large number of mosquitoes and absorb many infectious bites.

What does this mean for control? When *R*_0_ is in the thousands, eliminating malaria may seem impossible. If transmission from the humans who are bitten the most can be targeted, however, local elimination can still be within reach.

## Abstract 3

A new mathematical approach applied to 121 human populations infected with malaria revises the basic reproductive number--an indication of the transmission intensity--up by an order of magnitude, with serious implications for effective malaria control.

## Introduction

Each year, Plasmodium falciparum causes approximately 515 million clinical malaria cases [1] and over one million deaths [2,3]. Most malaria-related mortality and a large fraction of malaria cases occur in sub-Saharan Africa, where transmission can be very intense [4]. Strategic planning for malaria control should consider the transmission intensity of malaria, which is described by several parasitological and entomological indices (Table 1). The intensity of malaria transmission affects most aspects of malaria epidemiology and control, including the age at first infection, the fraction of a population that is infected (i.e., the parasite rate [PR]), the frequency and type of disease syndromes, the incidence of severe disease, the development and loss of functional immunity (i.e., immunity that reduces the frequency and severity of clinical symptoms), total malaria mortality, and the expected outcome of malaria control [4–8]. Good estimates of malaria transmission intensity are therefore necessary to compare and interpret malaria interventions conducted in different places and times and to objectively evaluate options for malaria control.

**Table 1:**
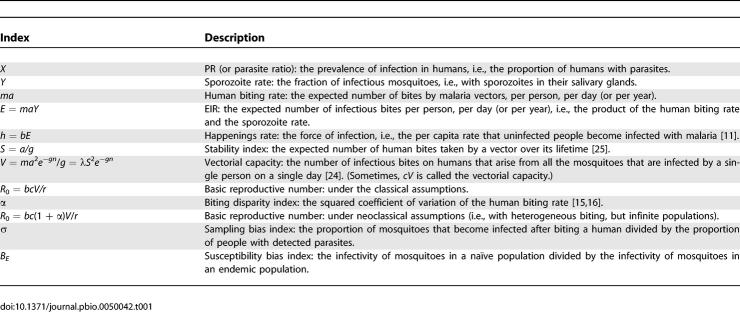
Indices of Malaria Transmission

The basic reproductive number, *R*_0_, has played a central role in epidemiological theory for malaria and other infectious diseases because it provides an index of transmission intensity and establishes threshold criteria. *R*_0_ is generally defined as the expected number of hosts who would be infected after one generation of the parasite by a single infectious person who had been introduced into an otherwise naïve population [9,10]. If *R*_0_ is greater than one, the number of people infected by the parasite increases, and if *R*_0_ is less than one, that number declines. Thus, if sustained disease control reduces transmission intensity by a factor that exceeds *R*_0_, the parasite will eventually be eliminated. Alternatively, the fraction of a population that would need to be protected to confer “herd immunity” and interrupt transmission is 1 − 1/*R*_0_.

The classic formula for *R*_0_ is based on a quantitative description of the P. falciparum life cycle [11,12] (Figure 1). It assumes that human populations are effectively infinite and that all humans are bitten at the same rate, but human populations are finite and some people are bitten by vectors more than others [13,14]. In infinite human populations, heterogeneous biting increases *R*_0_ because those humans who are bitten most are also most likely to become infected and then, by infecting a large number of mosquitoes, to amplify transmission [15,16]. Thus, in infinite human populations, the classic formulas underestimate *R*_0_.

**Figure 1:**
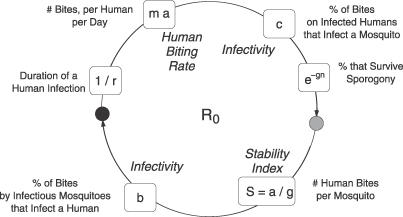
The Life Cycle Model and *R*_0_ The basic reproductive number, *R*_0_, is derived by computing the expected number of vertebrate hosts or vectors that would be infected through one complete generation of the parasite by a single infected mosquito or a single infected human. The underlying mathematical model, by Ross [11] and Macdonald [12] and with a slight modification by Smith and McKenzie [27], is a quantitative description of the idealized life cycle. This diagram follows one by Macdonald et al. [23]. The parameters are described in Table 2.

**Table 2:**
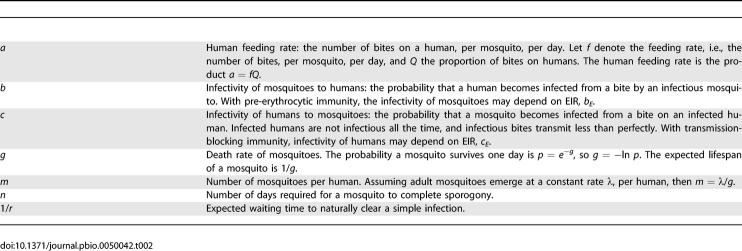
The Parameters

Classic and neoclassic (i.e., with heterogeneous biting) formulas for *R*_0_ describe idealized populations, where each infectious bite lands on a different host. In reality, some infectious bites land on previously infected hosts because malaria transmission is local. The spatial scale of malaria transmission is affected by vector ecology, especially the distribution of larval habitat and host-seeking behavior, human population density and distribution, and human movement [17,18]. Therefore, we reconsider *R*_0_ in finite human populations with heterogeneous biting, where some bites reinfect humans or mosquitoes. When the number of humans is not effectively infinite, what is the expected number of infected hosts or vectors after one complete generation of the parasite? How are these expectations changed when biting is heterogeneous, and what do these ideas imply about malaria control?

Because *R*_0_ is both an index of how well malaria spreads and a measure of the effort required to eliminate malaria, it would be the ideal index for strategic malaria control planning, but it has not been routinely recorded. Previous estimates of *R*_0_ were made with a variety of methods, and they have a limited spatial coverage. Since each method introduces different sources of potential error and bias, the estimates are not directly comparable [10]. One method estimates each parameter in the classic and neoclassic formulas [19,20]; this is rarely done because it is technically and logistically difficult. A second method is based on the rate of increase in the number of human cases during an epidemic in an uninfected and immunologically naïve population [21,22]. Obviously, this method has limited application in most African populations, where a substantial fraction of people harbor malaria infections. Equilibrium methods, originally suggested by Macdonald and colleagues [23] (see Dietz [10] for a review), rely on mathematical models that describe the relations between *R*_0_ and the population at the steady state. The terms of *R*_0_ are rearranged into a set of indices that can be measured in populations where malaria is endemic, so they are most broadly applicable.

Here, we introduce new equilibrium methods for estimating *R*_0_ that consider heterogeneous biting and factors that introduce a bias, such as sampling issues and immunity. We have used these new ideas to estimate *R*_0_ for 121 African populations. These estimates are based on a common methodology and have a continental spatial coverage, so they provide a more useful index of malaria transmission than previous attempts, and one that is suitable for strategic planning for malaria control.

## Results

### Estimating *R*_0_

Our estimates of *R*_0_ are based on two more commonly measured indices called the entomological inoculation rate (EIR) (*E* in equations), which is the average number of infectious bites received by a person in a year, and the PR (also called the parasite ratio) (*X* in equations), which is the prevalence of malaria infection in humans. Like other equilibrium methods, our method relies on mathematical models that define the steady state relationships between indices and parameters; these are the EIR, the PR, the vectorial capacity, *V,* which measures the number of infectious bites that arise from all the mosquitoes that are infected by a single infectious person on a single day [24], the infectivity of humans to mosquitoes, *c,* and the stability index, *S,* which measures the number of human bites taken by a vector during its lifetime [25]. The classical parameters and several malaria transmission indices are described in Tables 1 and 2. At the equilibrium, the relationship between these indices is given by a simple formula (Methods):

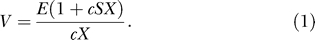
A simple relationship exists between *R*_0_ and vectorial capacity. *R*_0_ sums vectorial capacity, discounted for imperfect transmission efficiency, over the average infectious period [26,27]. In a population with heterogeneous biting, where the squared coefficient of variation in biting rates is α, *R*_0_ is larger by the factor 1 + α, because the humans who are bitten most amplify transmission [15,16]; we call α the index of biting disparity. The relationship between *R*_0_, vectorial capacity, and the other indices is given by the formula

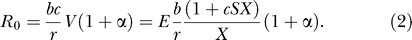
These formulas are based on the classic assumptions: mosquito lifespan and the duration of human infections are assumed to be exponentially distributed, and *R*_0_ is computed for a single parasite type (for a longer discussion of the assumptions, see the Methods).

Using equation 2, estimates of annual EIR and PR from studies of 121 African populations [3], and parameter estimates from other studies, we generated 121 estimates of *R*_0_ (Figure 2). Parameter estimates for *b*/*r* and α were taken from 91 of these studies that included only children less than 15 y old [14]. Published estimates of the stability index range from less than one up to five [9,28]; we use the estimate *S ≈* 1, at the low end of published studies. For the infectivity, we use the value *c* = 0.5, a number that agrees with estimates from direct-feeding experiments [29].

**Figure 2:**
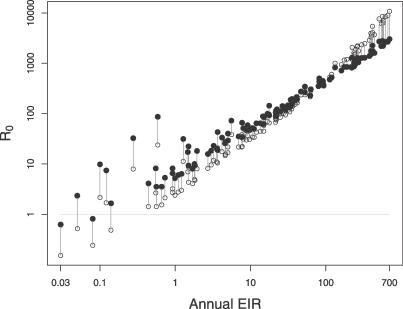
*R*_0_ Estimates for 121 African Populations Here, we show two different sets of estimates, plotted as a function of the estimated EIR. The first set of estimates assumes that none of the parameter estimates are biased by immunity or heterogeneous biting at the equilibrium (solid circles). The second set of estimates assumes that heterogeneous biting and transmission-blocking immunity bias parameters (open circles); σ is as illustrated by Figure 3. Corrections for this potential bias substantially increase the range of *R*_0_ estimates.

**Figure 3:**
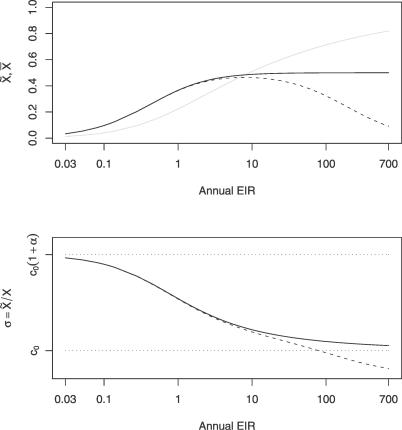
The Index of Sampling Bias, σ (Top) The PR (grey line) rises monotonically with EIR. The fraction of mosquitoes that become infected after biting a human, *X~*, is initially higher than the PR because of heterogeneous biting, but at high EIR, PR continues to rise while *X~* remains flat without transmission-blocking immunity (solid black line) or declines with it (dashed line). (Bottom) Without transmission-blocking immunity, the index of sampling bias, σ, declines from near *c*_0_(1 + α) to *c*_0_ (solid black line). At low EIR, the estimates of PR from a well-designed study underestimate the probability a mosquito becomes infected. At high EIR, without transmission-blocking immunity, this bias becomes insignificant. At high EIR, with transmission-blocking immunity, the PR in children substantially overestimates infectivity (dashed line). These graphs assume heterogeneous biting and model transmission-blocking immunity as in equation 20, with γ = 0.001 (Methods).

The *R*_0_ estimates range from near one to more than 3,000. The median was 115 and the interquartile range was 30−815. These values are consistent with previous estimates, including one estimate of 1,600 [20] in Mngeza, in northwest Tanzania, and another of 2,000–5,000 [19] in Lira township, in central Uganda. Had these studies considered heterogeneous biting, they would have exceeded our highest estimates.

In an area around Madang, Papua New Guinea, where entomological surveys have shown that annual EIR is approximately 150 [30], and where our methods would suggest that *R*_0_ is larger than 500, an estimate based on age seroprevalence was *R*_0_ ≈ 7. The biological basis for the large discrepancy remains unresolved; one possibility is the strain theory of transmission [31].

### Immunity and Sampling Bias

Equilibrium methods for estimating *R*_0_ are based on the simple assumptions of mathematical models; the difference between these simple assumptions and variance in real populations can introduce a large bias. When biting rates are heterogeneous, for example, mosquitoes bite infected humans at a different frequency than when humans are sampled in a study. Thus, PR may be a biased measure of the probability a mosquito becomes infected after biting a human. In addition, the intensity of transmission at equilibrium may be lower than it would be in that same population without immunity; immunity would reduce the infectivity of humans to mosquitoes (i.e., transmission-blocking immunity) [29,32], or mosquitoes to humans (i.e., by clearing an infection before the stages that infect red blood cells develop). We have derived new formulas that consider these potential sources of bias, and we use them to modify the previous estimates of *R*_0_.

When biting is heterogeneous and when there is some transmission-blocking immunity, it is necessary to introduce a new term called the sampling bias index, σ, that estimates the bias introduced by assuming that the fraction of mosquitoes that would become infected after biting a human is proportional to PR. σ is the ratio of two proportions. The numerator is the proportion of mosquitoes that become infected after biting a human, in a population at equilibrium; it is determined by EIR, by the index of biting disparity (Table 1), and by the level of transmission-blocking immunity. The denominator is the estimated PR, the proportion of humans that test positive in a study (Methods). Thus, the parameter σ encompasses several complex and poorly quantified processes, including differences in the way that human populations are “sampled” by mosquitoes and scientists, sporadic production of the infectious sexual stages during an infection (PR is an estimate of the prevalence of the noninfectious asexual stages), the reduced infectivity of humans to mosquitoes following the development of transmission-blocking immunity, and the sensitivity of the method used to detect parasites in humans.

When infectivity is estimated in a population where malaria is endemic and where there is some degree of immunity, the average infectivity of mosquitoes and humans, denoted *b_E_* and *c_E_,* respectively, may vary with EIR. The relevant parameters in the formula for *R*_0_ are taken from populations without immunity, so infectivity estimates would be from naïve populations, *b*_0_ and *c*_0_. The bias introduced by transmission-blocking immunity is included in σ. A correction for infections that are cleared before patency (i.e., before the stages that infect red blood cells are detected) is found by multiplying the formulas for *R*_0_ by the term *B_E_ = b*_0_/*b_E_,* which we call the susceptibility bias index.

Thus, we have a new formula for *R*_0_:

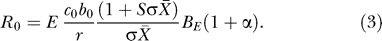
For the same 121 estimates of annual EIR and PR, we generated new estimates of *R*_0_ based on different assumptions about σ and *B_E_* (Figure 2). The original estimates effectively assumed that PR is a constant and unbiased index of infectivity (i.e., σ = 1) and that our estimates of susceptibility were not biased (i.e., *B_E_* = 1).

Our analysis suggests that σ is a complicated function of EIR (Figure 3; Methods). At low EIR (less than ten per year), mosquitoes sample infected individuals more efficiently than a stratified random sample of the population, so estimates of PR are biased by a factor that equals the product of infectivity and the amplification from heterogeneous biting, i.e., σ ≈ *c*_0_(1 + α). At moderate to high EIR (10–700 per year), transmission-blocking immunity reduces the average infectivity of infectious humans to mosquitoes, and since bites on those who have the most immunity account for a large fraction of bites, PR severely overestimates infectivity at high EIR.

When we assumed that transmission-blocking immunity develops, as illustrated in Figure 3, estimates of *R*_0_ ranged from below one to nearly 11,000, with a median of 86 and an interquartile range of 15–1,000.

### *R*_0_ in Finite Human Populations

The extremely high estimates of *R*_0_ raise the question of this index's interpretation in finite human populations; when *R*_0_ exceeds the number of humans, what does *R*_0_ actually describe? To interpret *R*_0_, we simulated transmission in small well-mixed human populations of size *H* through one complete parasite generation with heterogeneous and homogeneous biting (Figure 4; Methods). Let *R*_0_(*H*) denote the expected number of humans who could trace an infection back to one human, and *Z*_0_(*H*) the expected number of mosquitoes who could trace an infection back to one mosquito. (To clarify our notation, *R*_0_ is synonymous with *R*_0_(∞), so when population sizes are effectively infinite, *R*_0_ = *R*_0_(∞) = *Z*_0_(∞).) *R*_0_, *R*_0_(*H*), and *Z*_0_(*H*) can differ, depending on the host population size. When these three indices don't differ, the assumption that populations are effectively infinite is reasonably good. When they differ by more than 10%, we call the populations “small.” Small populations are defined by *R*_0_ and *H,* as well as the index of biting disparity, α, and the stability index, *S*.

**Figure 4:**
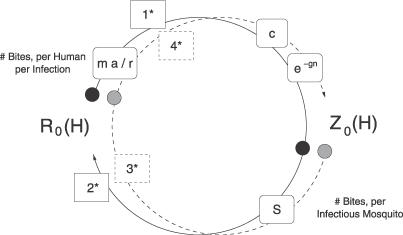
*R*_0_, *R*_0_(*H*), and *Z*_0_(*H*) in Finite Populations In finite populations, the number of different hosts infected through one complete generation of the parasite differs when the counting starts with humans, *R*_0_(*H*) (black circles, solid line), or with mosquitoes, *Z*_0_(*H*) (grey circles, dashed line), because of the different proportion of reinfected humans and mosquitoes (represented by boxes 1–4 with asterisks). These expectations are computed with heterogeneous biting, where individual biting rates differ from the average by the factor *s_i_,* called the biting weight (Methods). Box 1: for humans, a fraction of bites come from mosquitoes that were already infected (≈*H*/[*H* + *cS*]). Box 2: when these bites arrive back on a finite human population, they are distributed among the humans; some humans are bitten many times. The incidence of repeat infection is higher when *R*_0_ exceeds *H*. Box 3: starting with a single infectious mosquito, some fraction of humans become infected (less than *bS*), possibly more than once. Box 4: this affects the number of mosquitoes that are reinfected from biting the humans infected by a single mosquito (less than *H*/[*H* + *bcS* ^2^]). Explicit formulas are given in the Methods.

When the size of the human population was small and malaria transmission was very intense, *R*_0_(*H*) was limited by the number of humans; obviously, *R*_0_(*H*) ≤ *H*. If every human received exactly the same number of bites, some of them would have remained uninfected, by chance. With stochastic biting, there would be some variance in the number of bites received by each individual, even if the expected biting rates were uniformly distributed. Since humans received multiple bites, this tended to increase the proportion of bites that were absorbed by already infected humans, thereby reducing *R*_0_(*H*).

When human population sizes were effectively infinite, each infectious bite landed on a different human. In finite populations, heterogeneous biting amplifies transmission, as measured by *Z*_0_(*H*), just as it does for infinite populations, because those who are bitten most infect a large number of mosquitoes [15,16]. Surprisingly, heterogeneous biting reduced *R*_0_(*H*) below the expected number for homogeneous biting, especially when *R*_0_ was large and *H* was low (Figure 5). Heterogeneous biting reduced *R*_0_(*H*), i.e., the 20% of individuals who were bitten most also absorbed 80% of the infectious bites. Thus, a larger fraction of infectious bites were “reinfection” events; the transmission amplification associated with heterogeneous biting was nullified by a “superabsorbing” effect when those same individuals received most of the infectious bites.

**Figure 5:**
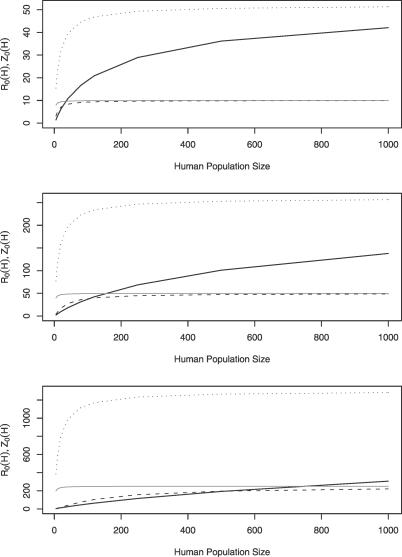
*R*_0_(*H*) and *Z*_0_(*H*) in Finite Populations with Heterogeneous and Uniform Biting at Three Biting Intensities The three biting intensities shown are for an *R*_0_ for homogenous biting equal to 10 (top), 50 (middle), and 250 (bottom). *R*_0_(*H*) rises slowly to *R*_0_, as a function of *H,* whether biting rates are heterogeneous (solid black lines) or uniform (dashed lines). Surprisingly, *R*_0_(*H*) for heterogeneous biting is lower than that for uniform biting, especially when *H* is low and *R*_0_ is high. By contrast, *Z*_0_(*H*) rises rapidly to *R*_0_ as a function of human population size, *H,* when biting rates are heterogeneous (dotted lines) or completely uniform (grey lines). These effects occur at population sizes well below those where the transmission-reducing effects of urbanization are evident [3].

The range of human population sizes that would be considered “small” differed for *Z*_0_(*H*) and *R*_0_(*H*) (Figure 5). *Z*_0_(*H*) rises to *R*_0_ much more rapidly than does *R*_0_(*H*), when considered as a function of human population size, *H*. Some mosquitoes become infected and return to bite the same human again; reinfection of mosquitoes affects both *R*_0_(*H*) and *Z*_0_(*H*). The fraction of mosquitoes that are reinfected depends mainly on the stability index, *S,* the index of biting disparity, α, and human population size. For reasonable estimates of *S* and α, *Z*_0_(*H*) approaches *R*_0_ when the neighborhood includes less than 100 humans (Methods).

“Small” for *R*_0_(*H*) depends on the ratio of *R*_0_ to *H* and the index of biting disparity. Some reinfection of mosquitoes does reduce *R*_0_(*H*), but this is a relatively unimportant effect for *H* > 25 (Methods). The much larger effect is reinfection of humans. Obviously, when *R*_0_ and *H* are of comparable size, repeat infection of humans substantially reduces *R*_0_(*H*), but when the human population is several times larger than *R*_0_, *R*_0_(*H*) ≈ *R*_0_, because very few people receive multiple bites. As a rule of thumb, *R*_0_(*H*) approaches *R*_0_ when *H* > 2*R*_0_. When 20% of people get 80% of the bites, the two measures are not close to one another until human population sizes are much larger: *R*_0_(*H*) ≈ *R*_0_ when *H* > 100*R*_0_.

The asymmetry between *R*_0_(*H*) and *Z*_0_(*H*) as a function of *R*_0_ and *H* arises because of the large difference in the number of humans infected by each mosquito and the number of mosquitoes infected by each human. Mosquitoes have short lives, typically 1–2 wk. The expected number of humans infected per mosquito—*cS* by our assumptions—is typically much less than three. The infectious period in humans, by contrast, stretches out over several months. The number of mosquitoes that bite a human during that time can range upwards to several thousand, limited mainly by the ratio of mosquitoes to humans. The number of mosquitoes infected by a single human can be so large that it exceeds the number of humans available to be bitten. When a large number of bites are distributed back on a limited number of humans, a substantial fraction result in reinfection.

### Control in Finite Populations

The large range of *R*_0_ estimates suggests that malaria control presents a variable challenge across Africa. At low transmission intensities, local elimination of malaria might be a practical goal. At the highest transmission intensities, classic theory suggests that transmission would need to be reduced by a factor of thousands, or that greater than 99% of hosts would need to be protected from infection. The amplification asymmetry that defines the relationship between *R*_0_, *H, R*_0_(*H*), and *Z*_0_(*H*) suggests that malaria control measures set different targets depending on the control method deployed. Here, we consider the implications of the extreme variation in *R*_0_ for control in finite populations with heterogeneous biting, where a few humans might account for a very large fraction of all infectious bites. In such populations, control measures that target those who are bitten most will tend to disproportionately reduce transmission. To explore these ideas, we simulated malaria control.

Because of differences in the way that control measures scale with human population size and alter transmission, we considered three categories of malaria control: host-based, vector-based, and mixed. Host-based methods, including antimalarial drugs or vaccines, reduce or completely neutralize transmission from hosts. Vector-based methods target vector populations in a general way: they lower the intensity of malaria transmission by reducing total vector density or adult lifespan. Mixed methods include insecticide-treated nets (ITNs) and indoor residual spraying (IRS). Like vector-based methods, they achieve their greatest effects by killing vectors, but like host-based methods, they are deployed around hosts to whom vectors are attracted.

Host-based methods include chemotherapy, chemoprophylaxis, and vaccines. Chemotherapy to clear infections would shorten the infectious period and reduce transmission. Obviously, case management does reduce the number of infectious individuals, but much larger reductions could be achieved through active detection of asymptomatically infected individuals followed by chemotherapy to clear infection. Since a person can become reinfected immediately after clearing an infection, more durable reductions would be achieved through chemoprophylaxis that completely neutralizes a host's ability to transmit. Similar effects would be also achieved through a vaccine that prevented infection, but no commercial vaccine for malaria is currently available or registered for public health use.

For perfect targeting, we simulated neutralizing that fraction of the individuals who were bitten most (Methods). With perfect targeting, herd immunity was achieved by neutralizing a relatively small fraction of hosts (Figure 6); neutralizing transmission from those who are bitten most makes the most of superabsorbing. The threshold population coverage required to confer herd immunity increased approximately linearly with the logarithm of *R*_0_, rising from around 20% of the human population when *R*_0_ was 50, to 50% when *R*_0_ was 2,000, much lower than the 98% and 99.95% coverage predicted to be necessary assuming homogeneous biting in infinite populations. When hosts were randomly neutralized, much higher coverage was required to achieve herd immunity; classic theory provided a useful guide, although the coverage required to achieve herd immunity was slightly lower in small populations (Figure 6).

**Figure 6:**
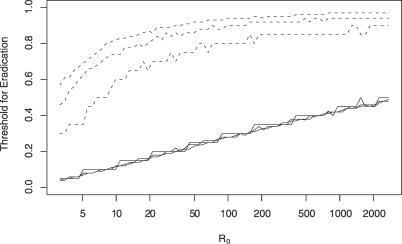
Achieving Herd Immunity with Random and Targeted Intervention This figure shows the relationship between *R*_0_ and the proportion of a population that must be neutralized through chemoprophylaxis or a vaccine if the intervention is perfectly targeted (solid lines) or random (dashed lines), such that *R*_0_ < 1. As the population size increases (from 20 to 50 to 100), the proportion that must be vaccinated increases for random intervention, but not for intervention targeted towards those bitten most.

Vector-based methods include mass spraying and other methods that target adult mosquitoes or larvae with pesticides or that reduce larval habitat. Our analysis suggests that *R*_0_ provides a fairly good measure of the factor by which transmission would have to be reduced by vector control to eliminate malaria. In very small populations, there is some repeat infection of mosquitoes regardless of *R*_0_, but as vector control reduces *R*_0_, repeat infection of humans becomes much less common.

Mixed methods merit a separate consideration from host- or vector-based methods because their success is often measured in terms of the proportion of hosts covered, and the effects on vector populations are more complicated than for purely vector-based control. Mixed methods reduce transmission from some hosts, but some vectors successfully feed despite ITNs or IRS, some mosquitoes are killed, and some vectors are repelled and attempt to bite again. We simulated targeted and random control with mixed methods (Methods; Figure 7). Like host-based methods, ITNs and IRS were very effective at reducing transmission when they were targeted, but the benefits also saturated after those who accounted for most of the bites were protected.

**Figure 7:**
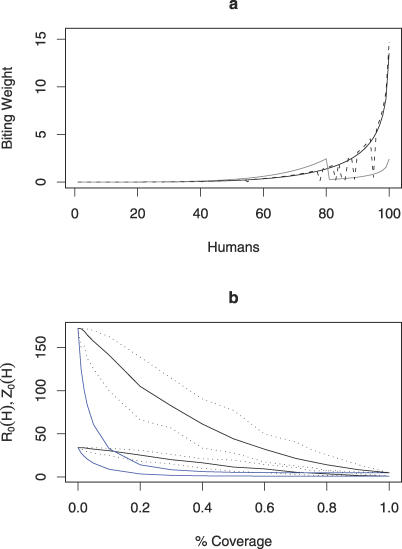
Changes in Transmission in Finite Populations with Heterogeneous Biting under Control by ITNs or IRS (A) In a population with 20% coverage, total biting decreases, but some bites are redistributed, so biting increases on those who are unprotected. The baseline biting weights (solid black line) are plotted, along with the comparable post-control biting weights after targeted (solid grey) and random (dashed) ITN distribution or IRS application. (B) ITNs or IRS reduce transmission more efficiently when they are targeted. (Here, *R*_0_ = 40, *R*_0_(*H*) ≈ 34, and *Z*_0_(*H*) ≈ 172.) For example, 10% targeted coverage (blue lines) and 70% random coverage (black lines; the solid line is the median and the dotted lines show the fifth and 95th quantiles) reduce *Z*_0_(*H*) (the lines that originate at 172) by about the same amount at the median. For these parameters, 100% coverage is required to reduce *R*_0_(*H*) below one, so for higher *R*_0_ values, 100% ITN or IRS coverage would be insufficient to eliminate malaria.

Despite the promise of enormous reductions in *R*_0_ through reductions in the lifespan of vectors [27], the total reductions in transmission from ITNs or IRS were limited. For the parameters we considered here, ITNs or IRS did not confer herd immunity, even with 100% coverage, for values of *R*_0_ well below our median estimate. The maximum reductions in transmission depended on the fraction of mosquitoes that were killed or deflected by ITNs or IRS, and there is substantial uncertainty about these parameters under field conditions. The maximum reductions were also affected by the stability index, a measure of an individual vector's transmission efficiency. The same level of transmission can be generated by a very large number of inefficient vectors, or a lesser number of efficient ones. ITNs and IRS were most effective at reducing transmission from very efficient vectors, i.e., vector populations with a high stability index. The lower the stability index, the lower the potential proportional reductions in transmission.

Since it was possible to achieve most of the reductions in transmission by targeting those who are bitten most, it might be possible to reduce costs by targeting. A side effect of ITNs or IRS was that the deflected bites were redistributed, so biting increased on those members of the population who were not protected (Figure 7). ITNs and IRS lower the risk of infection to unprotected individuals in the surrounding population by depleting vector populations, deflecting bites onto nonhuman hosts, or shortening vector lifespan. Despite the lower risk of infection overall, increased biting on unprotected hosts could increase their risk. Our analysis was focused on changes in *R*_0_(*H*) and *Z*_0_(*H*), so it did not explicitly consider the risk of infection, as measured by either EIR or PR. To evaluate these questions, a different sort of analysis would be required.

## Discussion

Estimates of the basic reproductive number (*R*_0_), the factor by which malaria transmission must be reduced through vector control in order to eliminate malaria, ranged from near one to more than 3,000 in a sample of 121 African populations. Revised estimates that considered other factors, such as sampling biases and immunity, that are potentially important but difficult to estimate suggest that the true range of *R*_0_ is even larger.

To put these *R*_0_ estimates in a broader context, the highest estimates of *R*_0_ are up to a thousand times higher than estimates of *R*_0_ for acute, directly transmitted infectious diseases [9]. However, *R*_0_ measures the number of new cases through one complete generation of the parasite, not the rate of increase in the number of cases per day. The time for malaria to complete one generation is more than 200 d [14]. During that time, diseases with an *R*_0_ of around two and a generation time of about 10 d, such as flu, for example, would have doubled 20 times in an effectively infinite host population and generated a million cases. Malaria generations overlap, so the number of expected cases after one disease generation would be higher than *R*_0_, but these extremely high *R*_0_ values do not necessarily represent a faster daily rate of increase for malaria compared to acute diseases with a much smaller *R*_0_. The goals that *R*_0_ values set for malaria control are high, but the longer generation times imply that there is more time for control.

### Strategic Planning

*R*_0_ is an important metric for strategic planning for malaria control because it helps to set priorities and define realistic expectations about the outcome of control. Despite the importance of *R*_0_, it has not been commonly estimated; the new estimates presented here increase the total number of published *R*_0_ estimates for malaria by an order of magnitude. The extremely large range of these *R*_0_ estimates suggests that a globally defined “one-size-fits-all” malaria control strategy would be inefficient. Where *R*_0_ is low, local elimination of malaria may be practical, even optimal. Where *R*_0_ is in the thousands, malaria may resist elimination even after heavy investments in multiple control measures [33]. In such populations, focused research to identify important aspects of local transmission would help to target control and achieve larger reductions.

Mathematical modeling and *R*_0_ provide a quantitative framework for strategic planning, one that can be modified to suit the local micro-epidemiology [34]. Important factors for control include the density and distribution of humans, the distribution of larval habitat, the vector species and their biting habits, and the seasonal patterns of transmission. Our analysis here suggests that the size of the local human population is also an important factor to consider, and that different methods may be effective (or cost-effective), depending on the distribution of humans and vectors.

Thus, an important factor in evaluating the success of malaria control is the spatial scale of malaria transmission, which is determined by several factors. Mosquito flight distances may be shorter when human blood meals are close to oviposition sites, so the spatial scale of transmission is codetermined by human population density, the distribution of humans and vector habitat, vector ecology, and vector behavior [17,18]. The spatial scale is also affected by the movement of humans. The formulas that link commonly measured entomological and parasitological indices to transmission intensity, and that correct these estimates for vector ecology and human population density, provide obvious opportunities for extensive mapping of malaria endemicity to help guide and rationalize control. These opportunities are explored in detail elsewhere [35].

### Targeting Intervention

The large reductions in transmission from targeting control are only possible if those who are bitten most can be identified, as has been done for some vector-borne diseases [36]. The feasibility of targeting depends strongly on the underlying causes of heterogeneous biting. Potential causes include mosquito aggregation around places where adult mosquitoes emerge [17] or vectors oviposit [18]; also, some components of breath and sweat [37] and dirty linen [38,39] make some humans inherently more attractive to mosquitoes [39,40]. Other causes of differential biting include the use of bed nets, protective clothing, and repellants [41], housing quality and design [42], pregnancy [43], alcohol consumption [44], body size [45], and defensive behavior [46]. With research, some of these may be exploited to identify and target those who are bitten most, and thereby improve malaria control.

One practical idea is to target those with clinical malaria and presumptively treat their families and nearest neighbors with efficacious antimalarial drugs with antigametocidal properties (i.e., that clear the infectious stages) [47] to clear infection and reduce the local reservoir. In low transmission areas, where a large fraction of new malaria infections result in clinical malaria, such targeting has demonstrably reduced transmission [48,49]. In high transmission areas, where a lower fraction of new cases result in clinical malaria, clinical malaria in young children may provide some indication of where drug treatment would be most effectively targeted. In such areas, the required reductions in transmission intensity are unlikely to be achieved by any single control measure. Where *R*_0_ exceeds a thousand, the additional widespread use of ITNs and supplementary targeted IRS may be required to achieve desired reductions in morbidity and mortality [33].

In small human populations, transmission may be effectively controlled by identifying those individuals who are most important for transmission and neutralizing their potential to transmit malaria. For example, consider an island that has only a few people, but many vectors. If one additional person came ashore infected with malaria, an epidemic would tend to ensue, if *R*_0_(*H*) > 1. It may not be possible to control the epidemic with ITNs (i.e., because *Z*_0_(*H*) ≫ 1), but malaria could be rapidly eliminated by clearing the infection from these individuals and preventing new infections with chemoprophylaxis. In large human populations, malaria could be controlled by targeting the same fraction of humans, but this might represent a very large number of people, so the costs may differ dramatically relative to control measures in small populations.

Our analysis suggests that *R*_0_ provides a reasonably good estimate of the reductions in transmission intensity that would be required to eliminate malaria through vector-based control. Obviously, the decision to invest in vector-based control depends on many considerations. Like heterogeneous biting, the heterogeneous distribution of adult emergence rates from larval habitats would affect the benefits of larval control. If most of the adult mosquitoes could be eliminated by removing a few larval breeding sites, targeting larval habitats might produce a large gain for little effort. In the extreme case, if all the mosquitoes emerged from a well, the easiest solution might be to cover the well. Since the benefits are related to the number of humans who would benefit, vector-based control will be more cost-effective when there are many humans. In large, urban populations, it might be more cost-effective to target vector populations for control, because of the simple fact that there is much less area to treat and many more people who benefit [50].

### Reiterating Basic Principles

The effectiveness of various malaria control methods depends on the context of local transmission, but several general principles derived from the classic modeling efforts are germane. First, since the infectious period for malaria can be extremely long and a substantial fraction of the P. falciparum reservoir resides in asymptomatic cases, the infectious period can be shortened and the reservoir of parasites reduced by the use of antimalarial drugs. Thus, effective antimalarial drugs can be important tools for malaria control as well as for treating clinical malaria, although this does raise concerns about the spread of resistance. Second, although the intensity of malaria transmission is exquisitely sensitive to the mortality rate of adult mosquitoes [27], potential reductions in transmission intensity via manipulations of this parameter are limited by the fact that ITNs and IRS are not completely efficient; the maximum benefits depend on the fraction of mosquitoes that are killed or repelled and on aspects of the vector populations, especially the stability index. Because most of the reductions in transmission come from protecting a few humans, it is far more important to improve the killing effects of ITNs or IRS around those who are bitten most than to improve coverage on those who are bitten least; however, complete coverage and improved killing effects may be necessary to reach control goals. Finally, when host population sizes are small or transmission is very localized, targeted neutralization may be an extremely effective way to protect other people in the community from getting malaria. In some places, vector control may be an effective and cost-effective way to reduce the burden of malaria [2], and it has had some historical success [51], but it may not be cost-effective everywhere.

In some of the African populations described here, where malaria transmission is very intense, no single control measure will be sufficient. Nevertheless, if the suite of interventions appropriate for the transmission regime could be implemented at the appropriate targeted scale in many malaria-endemic nations, the malaria-related millennium development goals could be achieved well before an effective vaccine is available. Clarifying the optimal mix of interventions and how these can be mapped and optimally targeted at scale thus remains an important direction for our collective future research.

## Materials and Methods

### Estimate 1: The life-cycle model.

Ross developed and Macdonald modified a mathematical model for the transmission of a vector-borne disease that is a simplified quantitative description of the parasite life cycle [11,12]. The parameter names, following Macdonald's notation, are given in Table 2. The life-cycle model tracks the fraction of infected humans, *X,* and the fraction of infectious mosquitoes, *Y,* over time:

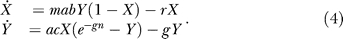
In this system of equations, the parasite persists if *R*_0_ > 1, where

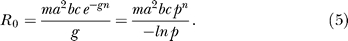
If *R*_0_ > 1, the equilibria are given by the expressions

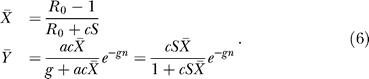
Since the average mosquito lifespan is short (i.e., 1/*g* ≈ 10–20 d), but the malaria infections in humans last months (i.e., *b/r ≈* 170 d [14]), the proportion of infectious mosquitoes adjusts rapidly to the proportion of infectious humans, i.e., the sporozoite rate tracks PR when mosquito populations are constant (but see the discussions by Aron and May [52] and by Smith et al. [17]).

Thus, EIR is given by the formula

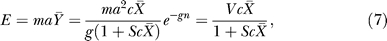
where *V* denotes vectorial capacity, following the original definition (see Table 2) [24]. Solving for *V,* we get

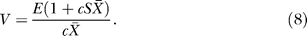
By our notation *R*_0_ = *bcV*/*r,* so we can compute *R*_0_ by solving for vectorial capacity:

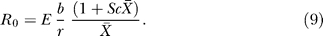



Dietz [15] and Dye and Hasibeder [16] have demonstrated that *R*_0_ is higher because of heterogeneous biting:

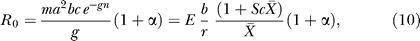
where α is the squared coefficient of variation of the human biting rate.

In these equations, mortality during sporogony is counted, but the delay for sporogony is not [17]. These equations give expressions for *R*_0_ and equilibria, *X̄*and *Ȳ*, that are consistent with the simple assumptions of the classic model. These equations differ slightly from those given by Anderson and May, who write 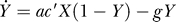 [9], but the equilibrium 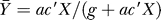 would not be consistent with the standard assumptions when mortality during sporogony is incorporated by setting *c*′ = *ce* ^−*gn*^ [27]. Closely related delay equations are given by Aron and May [52]. An alternative approach incorporating a realistic incubation period was modeled by Smith et al. [17]. All these models assume constant per capita mortality for mosquitoes, and so they ignore important factors such as temperature-dependent mortality and senescence.

Macdonald et al.'s equilibrium method estimates *R*_0_ from the force of infection [23]; usually, these estimates of *h* are based on the change in PR with age in cross-sectional surveys:

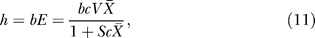
so

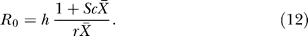



### Superinfection.

The estimates of *b/r* and α come from a nonlinear regression analysis using a model with superinfection (i.e., multiple infections) [14]. Here, the connection between that model and the life-cycle model is explained.

A generalized form of the life-cycle model tracks the fraction of the human population with some number of parasite “broods” [53–55], denoted *i*. New broods are introduced by new infections at the happenings rate, which might depend on the number of broods present, *h_i_,* and these broods are cleared naturally, also depending on the number of broods present, ρ*_i_.* The change in the fraction of uninfected humans is described by an equation:

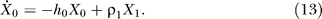
The change in the fraction of humans that are infected with *i* broods is given by

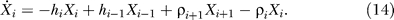
This is an extremely general formulation of a model for infection, although the idea of a “brood” remains poorly defined. For different assumptions about *h_i_* and ρ*_i_,* and for explicit assumptions about transmission of different broods by mosquitoes, it is possible to generate a very large number of models for infection in humans; some of these have been worked out by Dietz [56].

With a single brood, the dynamics reduce to the classical formulation. If there are a very large (effectively infinite) number of broods, then the force of infection is constant, *h_i_* = *bE*. For an infinite number of broods that clear independently, i.e., ρ*_i_* = *ir,* the distribution of brood number at equilibrium is Poisson with mean *bE/r* [55], and the fraction infected is given by

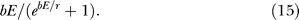
These estimates of *R*_0_ are based on Smith et al.'s estimate of *b/r,* which is based on the infinite brood and independent clearance model [14]. In turn, the formulas for *R*_0_ consider the invasion of a population by a single brood.

### Estimate 2: Immunity and heterogeneous biting.

The probability that a mosquito becomes infected, per bite, in the life-cycle model is denoted *cX*. In reality, transmission-blocking immunity and heterogeneous biting skew the probability that a mosquito becomes infected, per bite. Let *X~* denote the probability that a mosquito becomes infected after biting a human (i.e., in the life-cycle model *X~* = *cX*); then, infection in mosquitoes follows the equation

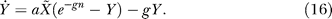
Following similar arguments as before, we get that vectorial capacity is given by the formula

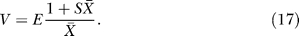



Because of transmission-blocking immunity, infectivity of humans declines as a function of EIR, denoted *c_E_*. Similarly, immunity at the liver stage affects the average infectivity of mosquitoes, denoted *b_E_*. Since *R*_0_ is defined for naïve populations, the formulas are based on infectivity in naïve hosts, *c*_0_ and *b*_0_. Following similar arguments as before:

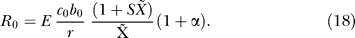
Since our estimate of *b/r* may actually be an estimate of *b_E_/r,* we need to correct the estimate by the ratio *B_E_* = *b*_0_/*b_E_*.

The bias introduced by transmission-blocking immunity depends implicitly on heterogeneous biting. With heterogeneous biting, mosquitoes bite individuals with index *s* at the rate *sE; s* is called a biting weight. Let *X*(*s*) denote the fraction of individuals with biting weight *s* that are infected, and let Γ(*s*,α) be the fraction of the population that has index *s* [14]. Finally, let *c*(*sE*) denote the average infectivity of humans who have a personal expected biting rate, *sE*. It follows that the probability a mosquito becomes infected after biting a human is

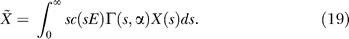
We let *c*(*sE*) = *c*_0_ *e* ^−γ*sE*^ *,* so that, because of the development of transmission-blocking immunity, infectivity declines in those who are bitten most. Using the Γ distribution and the equations for superinfection, as in [14], equation 19 can be solved:

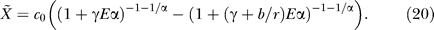
Similarly, prevalence, Ȳ, is given by [14]:

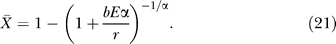
We assume that a well-designed study would estimate Ȳ, while a mosquito sees *X~*.

The sampling bias index is 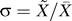 . Using this formula, we can estimate *R*_0_ as a function of EIR and PR:

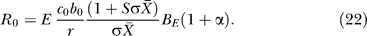
We note that when EIR is low, σ ≈ *c*_0_(1 + α), so this formula simplifies to the following:

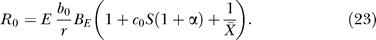



### Human to human in finite populations.

Here, we explore the interpretation of *R*_0_ in finite populations, of size *H*. This approach is motivated by the extremely high estimates of *R*_0_ (or *R*_0_(∞)), which in some cases may even exceed the local human population size. Here, *R*_0_(*H*) is defined as the expected number of different individual humans that can trace an infection back to a single human after one complete generation of the parasite, and *Z*_0_(*H*) is the number of mosquitoes that can trace an infection back to a single mosquito.

Mathematical approaches to *R*_0_ have evolved since Macdonald [12], and so have the definitions. We maintain the connection to Macdonald's original definition, in part, for historical continuity. Nowadays, *R*_0_ is computed using next-generation approaches [10,57]. By those definitions, the quantity that we compute is called 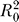 . Next-generation approaches are linearized approximations, and *R*_0_ is an eigenvalue associated with asymptotic growth rates. Our reevaluation of *R*_0_ is motivated by a different case—when *R*_0_ and *H* are of comparable size—so asymptotic growth rates are not our primary interest. Since *R*_0_(*H*) and *Z*_0_(*H*) differ, it is possible that *R*_0_(*H*) < 1, but *Z*_0_(*H*) > 1. In finite populations where *R*_0_(*H*) and *Z*_0_(*H*) are both near one, malaria would be likely to random walk to extinction, in any case.

To compute *R*_0_(*H*) or *Z*_0_(*H*) in heterogeneous populations, let *i* = 1...*H* index humans in a population, and let *mas_i_* denote their individual biting rates, where the distribution of biting weights, {*s*}, is constrained to have a mean of 1; 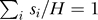 . The proportion of bites that land on the *i*th individual is therefore *s_i_*/*H*.

First, we compute the number of infected humans, per human. While infectious, the *i*th human receives *mas_i_/r* bites. Each mosquito biting the individual becomes infected with probability *c,* but some fraction of these mosquitoes return to bite the *i*th human again, so we need to discount multiple infection of mosquitoes. The fraction of bites on the *i*th human is *s_i_/H,* so a short time after the *i*th human has become infected, the fraction of mosquitoes that were already infected by that human is

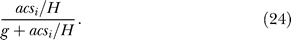
The proportion of those bites that infect a different mosquito is

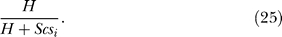
Note that more than 90% of bites are new infections when *H >* 9*cS,* so reinfection of mosquitoes is a relatively small effect when *H* > 25. Thereafter, the mosquito survives to become infectious with probability *ce^−gn^,* and then is expected to give *a/g* infectious bites.

Thus, the total number of infectious bites that arise from the *i*th human is

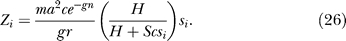
The *j*th human in that population is expected to be bitten at the rate *Z_i_s_j_/H,* and each bite causes an infection with probability *b*. Thus, the probability that the *j*th individual remains uninfected is

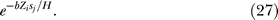
If the *i*th person is the index case, the expected number of infected humans is

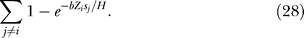
There are two reasonable expectations to be computed. First is the unweighted expectation:

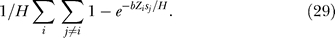
The second is the weighted expectation:

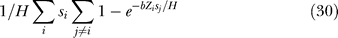
We prefer this second, weighted expectation because it reflects heterogeneous biting, because those who are bitten most are most likely to be the index case, and because in the infinite human population limit, it converges to the formula for *R*_0_ derived by Dietz [15] and Dye and Hasibeder [16]. Note that α is the squared CV of {*s*} and that 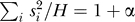 .

### Mosquito to mosquito in finite populations.

From a single infectious mosquito, the expected number of bites that produce an infection is *bS*. The probability that the *i*th person becomes infected is

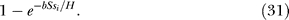
Thereafter, that person gets *mas_i_/r* bites before clearing an infection.

The number of infected humans is

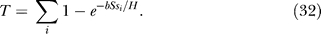
A fraction *c* of all bites infect uninfected mosquitoes. As before, some fraction of mosquitoes are already infected. We consider only those infected mosquitoes that can trace their infection back to the index mosquito, so following the previous argument, the fraction of mosquito infections that are not reinfections is 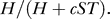


Thereafter, *e^−gn^* infected mosquitoes survive to become infectious.

Therefore, the total number of infectious mosquitoes per infectious mosquito is given by the formula

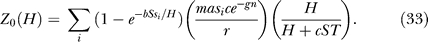
The fraction of newly infected mosquitoes increases rapidly as a function of *H*. In a very large population, *T* is less than *bS;* more than 90% of bites are new infections when *H* > 9*cST* > 9*bcS* ^2^.

### Simulated control in finite populations: Human-based methods.

When transmission from humans is neutralized by a perfect vaccine or by chemoprophylaxis, infected humans continue to absorb bites, but don't infect any mosquitoes. We construct a vector of length *H* where *V_j_ =* 0 if an individual is protected, and *V_j_ =* 1 otherwise. With targeted protection,

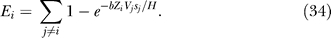
Note that *V_j_* appears in the exponent to account for bites on neutralized individuals. To compute *R*_0_(*H*) with neutralization, we compute the weighted expectation:

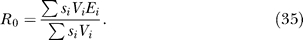
Here, *V_i_* removes protected individuals from the computation—if a person is protected, then it is not possible for him to be the index case, by assumption.

### Simulated control in finite populations: Pure mosquito-based methods.

After controlling vector populations, estimates of *R*_0_(*H*) and *Z*_0_(*H*) would be computed as before, but with different estimates of *m* or *g*. It is also possible that vector control would change the distribution of biting weights, but this is not a question that we have addressed here.

### Simulated control in finite populations: Mixed methods.

When humans are protected from infection by ITNs or by IRS, some fraction of the mosquitoes that attempt to bite a protected human are killed, and some fraction are diverted onto other hosts. To model both effects, we assume that the biting weights describe the probability of finding a host during each attempt, that a fraction of biting attempts on protected humans kill the mosquito each visit (denoted δ), that a fraction of mosquitoes successfully feed (ψ), and that those mosquitoes that neither die nor successfully feed fly off to begin a new search. Of these, a fraction *Q* finds a human, again. Let *N* denote the set of people who are protected, then the fraction of visits that find a protected human is 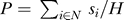 .

We ignore the delay required to find another host, and assume that the vectors instantaneously reassort themselves onto hosts until they have either died or successfully fed. The fraction of mosquitoes that die is δ*P* at the first attempt, plus δ*P* times all those who failed to feed the first time and again find a protected human, and so on:

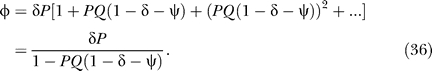
Thus, φ is the fraction of human feeding attempts by vectors that result in mosquito death. With ITN use, the mosquito death rate increases to *g*′ = *g* + φ*a*. By a similar argument, the feeding rate on the *i*th protected host is

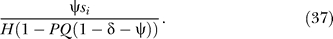
And the proportion of bites on the *j*th unprotected hosts increases to

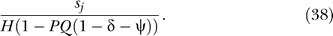
In a finite population, we compute *R*_0_(*H*) and *Z*_0_(*H*) as before, with new parameters describing human feeding, mosquito mortality, and biting weights (which may not sum to one). Obviously, the success of ITNs depends on the baseline parameters, *Q*, δ, and ψ. Here, we simulate control for *Q =* 0.9, δ = 0.3, and ψ = 0.2.

## Abbreviations

EIR: entomological inoculation rate
IRS: indoor residual spraying
ITN: insecticide-treated net
PR: parasite rate

## References

[pbio-0050042-b001] Snow RW, Guerra CA, Noor AM, Myint HY, Hay SI (2005). The global distribution of clinical episodes of Plasmodium falciparum malaria. Nature.

[pbio-0050042-b002] Snow RW, Omumbo JA, Jamison DT, Feachem RG, Makgoba MW, Bos ER, Baingana FK (2006). Malaria. Disease and mortality in sub-Saharan Africa, 2nd edition.

[pbio-0050042-b003] Hay SI, Guerra CA, Tatem AJ, Atkinson PM, Snow RW (2005). Urbanization, malaria transmission, and disease burden in Africa. Nat Rev Microbiol.

[pbio-0050042-b004] Snow RW, Omumbo JA, Lowe B, Molyneux CS, Obiero JO (1997). Relation between severe malaria morbidity in children and level of Plasmodium falciparum transmission in Africa. Lancet.

[pbio-0050042-b005] Marsh K, Snow RW (1999). Malaria transmission and morbidity. Parassitologia.

[pbio-0050042-b006] Snow RW, Marsh K (2002). The consequences of reducing transmission of Plasmodium falciparum in Africa. Adv Parasitol.

[pbio-0050042-b007] Struik SS, Riley EM (2004). Does malaria suffer from lack of memory?. Immunol Rev.

[pbio-0050042-b008] Reyburn H, Mbatia R, Drakeley C, Bruce J, Carneiro I (2005). Association of transmission intensity and age with clinical manifestations and case fatality of severe Plasmodium falciparum malaria. JAMA.

[pbio-0050042-b009] Anderson RM, May RM (1991). Infectious diseases of humans.

[pbio-0050042-b010] Dietz K (1993). The estimation of the basic reproduction number for infectious diseases. Stat Methods Med Res.

[pbio-0050042-b011] Ross R (1910). The prevention of malaria.

[pbio-0050042-b012] Macdonald G (1957). The epidemiology and control of malaria.

[pbio-0050042-b013] Woolhouse ME, Dye C, Etard JF, Smith T, Charlwood JD (1997). Heterogeneities in the transmission of infectious agents: Implications for the design of control programs. Proc Natl Acad Sci U S A.

[pbio-0050042-b014] Smith DL, Dushoff J, Snow RW, Hay SI (2005). The entomological inoculation rate and its relation to the prevalence of Plasmodium falciparum infection in African children. Nature.

[pbio-0050042-b015] Dietz K (1980). Models for vector-borne parasitic diseases. Lect Notes Biomath.

[pbio-0050042-b016] Dye C, Hasibeder G (1986). Population dynamics of mosquito-borne disease: Effects of flies which bite some people more frequently than others. Trans R Soc Trop Med Hyg.

[pbio-0050042-b017] Smith DL, Dushoff J, McKenzie FE (2004). The risk of a mosquito-borne infection in a heterogeneous environment. PLoS Biol.

[pbio-0050042-b018] Le Menach A, McKenzie FE, Flahault A, Smith DL (2005). The unexpected importance of mosquito oviposition behaviour for malaria: Non-productive larval habitats can be sources for malaria transmission. Malar J.

[pbio-0050042-b019] Davidson G, Draper CC (1953). Field studies on some of the basic factors concerned in the transmission of malaria. Trans R Soc Trop Med Hyg.

[pbio-0050042-b020] Davidson G (1955). Further studies of the basic factors concerned in the transmission of malaria. Trans R Soc Trop Med Hyg.

[pbio-0050042-b021] Macdonald G (1956). Theory of the eradication of malaria. Bull World Health Organ.

[pbio-0050042-b022] Freeman J, Laserson KF, Petralanda I, Spielman A (1999). Effect of chemotherapy on malaria transmission among Yanomami Amerindians: Simulated consequences of placebo treatment. Am J Trop Med Hyg.

[pbio-0050042-b023] Macdonald G, Cuellar CB, Foll CV (1968). The dynamics of malaria. Bull World Health Organ.

[pbio-0050042-b024] Garrett-Jones C (1964). Prognosis for interruption of malaria transmission through assessment of the mosquito's vectorial capacity. Nature.

[pbio-0050042-b025] Davidson G (1954). Estimation of the survival of *Anopheline* mosquitoes in nature. Nature.

[pbio-0050042-b026] Dye C (1986). Vectorial capacity: Must we measure all its components. Parasitol Today.

[pbio-0050042-b027] Smith DL, McKenzie FE (2004). Statics and dynamics of malaria infection in anopheles mosquitoes. Malar J.

[pbio-0050042-b028] Killeen GF, McKenzie FE, Foy BD, Schieffelin C, Billingsley PF (2000). A simplified model for predicting malaria entomologic inoculation rates based on entomologic and parasitologic parameters relevant to control. Am J Trop Med Hyg.

[pbio-0050042-b029] Githeko AK, Brandling-Bennett AD, Beier M, Atieli F, Owaga M (1992). The reservoir of Plasmodium falciparum malaria in a holoendemic area of western Kenya. Trans R Soc Trop Med Hyg.

[pbio-0050042-b030] Burkot TR, Graves PM, Paru R, Wirtz RA, Heywood PF (1988). Human malaria transmission studies in the Anopheles punctulatus complex in Papua New Guinea: Sporozoite rates, inoculation rates, and sporozoite densities. Am J Trop Med Hyg.

[pbio-0050042-b031] Gupta S, Trenholme K, Anderson RM, Day KP (1994). Antigenic diversity and the transmission dynamics of Plasmodium falciparum. Science.

[pbio-0050042-b032] Muirhead-Thomson RC (1954). Factors determining the true reservoir of infection of Plasmodium falciparum and Wuchereria bancrofti in a West African village. Trans R Soc Trop Med Hyg.

[pbio-0050042-b033] Molineaux L, Gramiccia G (1980). The Garki project: Research on the epidemiology and control of malaria in the Sudan savanna of West Africa.

[pbio-0050042-b034] Greenwood B (1989). The microepidemiology of malaria and its importance to malaria control. Trans R Soc Trop Med Hyg.

[pbio-0050042-b035] Hay SI, Snow RW (2006). The Malaria Atlas Project (MAP): Developing global maps of malaria risk. PLoS Med.

[pbio-0050042-b036] Perkins SE, Cattadori IM, Tagliapietra V, Rizzoli AP, Hudson PJ (2003). Empirical evidence for key hosts in persistence of a tick-borne disease. Int J Parasitol.

[pbio-0050042-b037] Mukabana WR, Takken W, Coe R, Knols BG (2002). Host-specific cues cause differential attractiveness of Kenyan men to the African malaria vector Anopheles gambiae. Malar J.

[pbio-0050042-b038] Knols BG (1996). On human odour, malaria mosquitoes, and limburger cheese. Lancet.

[pbio-0050042-b039] Murphy MW, Dunton RF, Perich MJ, Rowley WA (2001). Attraction of *Anopheles* (Diptera: culicidae) to volatile chemicals in Western Kenya. J Med Entomol.

[pbio-0050042-b040] Takken W, Knols BGJ (1999). Odor-mediated behavior of Afrotropical malaria mosquitoes. Annu Rev Entomol.

[pbio-0050042-b041] Srinivas G, Edwin Amalraj R, Dhanraj B (2005). The use of personal protection measures against malaria in an urban population. Public Health.

[pbio-0050042-b042] Lindsay SW, Snow RW (1988). The trouble with eaves; house entry by vectors of malaria. Trans R Soc Trop Med Hyg.

[pbio-0050042-b043] Ansell J, Hamilton KA, Pinder M, Walraven GE, Lindsay SW (2002). Short-range attractiveness of pregnant women to Anopheles gambiae mosquitoes. Trans R Soc Trop Med Hyg.

[pbio-0050042-b044] Shirai O, Tsuda T, Kitagawa S, Naitoh K, Seki T (2002). Alcohol ingestion stimulates mosquito attraction. J Am Mosq Control Assoc.

[pbio-0050042-b045] Port GR, Boreham PFL, Bryan JH (1980). The relationship of host size to feeding by mosquitoes of the Anopheles gambiae giles complex (Diptera: Culicidae). Bull Entomol Res.

[pbio-0050042-b046] Kelly DW (2001). Why are some people bitten more than others?. Trends Parasitol.

[pbio-0050042-b047] Sutherland CJ, Ord R, Dunyo S, Jawara M, Drakeley CJ (2005). Reduction of malaria transmission to *Anopheles* mosquitoes with a six-dose regimen of co-artemether. PLoS Med.

[pbio-0050042-b048] Nosten F, van Vugt M, Price R, Luxemburger C, Thway KL (2000). Effects of artesunate-mefloquine combination on incidence of Plasmodium falciparum malaria and mefloquine resistance in western Thailand: A prospective study. Lancet.

[pbio-0050042-b049] Barnes KI, Durrheim DN, Little F, Jackson A, Mehta U (2005). Effect of artemether-lumefantrine policy and improved vector control on malaria burden in KwaZulu–Natal, South Africa. PLoS Med.

[pbio-0050042-b050] Conteh L, Sharp BL, Streat E, Barreto A, Konar S (2004). The cost and cost-effectiveness of malaria vector control by residual insecticide house-spraying in southern Mozambique: A rural and urban analysis. Trop Med Int Health.

[pbio-0050042-b051] Killeen GF, Knols BG, Gu W (2003). Taking malaria transmission out of the bottle: Implications of mosquito dispersal for vector-control interventions. Lancet Infect Dis.

[pbio-0050042-b052] Aron JL, May RM, Anderson RM (1982). The population dynamics of malaria. Population dynamics and infectious disease.

[pbio-0050042-b053] Walton GA (1947). On the control of malaria in Freetown, Sierra Leone. I. Plasmodium falciparum and Anopheles gambiae in relation to malaria occurring in infants. Ann Trop Med Parasitol.

[pbio-0050042-b054] Dietz K, Molineaux L, Thomas A (1974). A malaria model tested in the African savannah. Bull World Health Organ.

[pbio-0050042-b055] Bailey NTJ (1982). The biomathematics of malaria.

[pbio-0050042-b056] Dietz K (1988). Density dependence in parasite transmission dynamics. Parasitol Today.

[pbio-0050042-b057] Diekmann O, Heesterbeek JAP, Metz JAJ (1990). On the definition and the computation of the basic reproduction ratio *R*
_0_ in models for infectious diseases in heterogeneous populations. J Math Biol.

